# Hearing Aid Service Models, Technology, and Patient Outcomes: A Randomized Clinical Trial

**DOI:** 10.1001/jamaoto.2025.1008

**Published:** 2025-05-15

**Authors:** Yu-Hsiang Wu, Elizabeth Stangl, Kjersten Branscome, Jacob Oleson, Todd Ricketts

**Affiliations:** 1 Department of Communication Sciences and Disorders, University of Iowa, Iowa City; 2 Department of Hearing and Speech Sciences, Vanderbilt University Medical Center, Nashville, Tennessee; 3 Department of Biostatistics, University of Iowa, Iowa City

## Abstract

**Importance:**

The poor affordability of hearing aids (HAs) limits their adoption. To justify higher costs, HAs fitted by audiologists (AUD service model) and high-end HAs should deliver better outcomes than over-the-counter (OTC) service models and low-end HAs.

**Objective:**

To determine the effect of HA service models (AUD, OTC, and a hybrid OTC+ model) and technology levels (high end and low end) on patient outcomes.

**Design, Setting, and Participants:**

This randomized clinical trial was conducted at the University of Iowa and Vanderbilt University Medical Center in research laboratories from February 2019 to December 2023 and included adults older than 55 years with mild to moderate hearing loss and no previous HA experience who were randomly assigned to 1 of 6 parallel groups, representing factorial combinations of 3 service models and 2 technology levels. The data were analyzed between January 2024 and March 2024.

**Interventions:**

The trial included 3 service models: AUD, in which audiologists fitted prescription HAs following best practices; OTC+, in which audiologists provided limited services for OTC HAs; and OTC, in which participants independently used OTC HAs. OTC HAs were simulated using prescription HAs. Two models of prescription HAs were used throughout the trial: a high-end HA with advanced features and a low-end HA.

**Main Outcomes and Measures:**

The primary outcome measure was the Glasgow Hearing Aid Benefit Profile (GHABP), which was administered using ecological momentary assessment (EMA). EMA-GHABP was conducted preintervention and throughout the seventh week postintervention.

**Results:**

A total of 245 participants completed the study (121 women [49.4%]; mean [SD] age, 67.7 [8.1] years). After controlling for preintervention scores, the postintervention EMA-GHABP global score (ranging from 1 to 5) for AUD was significantly higher (indicating better outcomes) than for OTC+ and OTC by 0.33 points (95% CI, 0.14-0.52) and 0.32 points (95% CI, 0.13-0.51), respectively. The difference between OTC+ and OTC was not significant (0.02 points, 95% CI, −0.21 to 0.18). Nevertheless, EMA-GHABP global scores for OTC+ and OTC were close to 4 points, indicating positive outcomes. The effect of technology level and interaction between service model and technology level were not significant.

**Conclusions and Relevance:**

The trial results suggest that while OTC+ and OTC were effective, they did not achieve the same outcomes as AUD. As high-end and low-end HAs yielded similar outcomes, support for the higher cost of high-end HAs was not identified for individuals with mild to moderate hearing loss.

**Trial Registration:**

ClinicalTrials.gov Identifier: NCT03579563

## Key Points

**Question:**

Do higher levels of hearing aid (HA) services and technology, which incur greater costs, lead to better patient outcomes?

**Findings:**

In this randomized clinical trial with 245 participants with mild to moderate hearing loss, the service model in which audiologists fitted prescription HAs following best practices yielded significantly better outcomes compared with 2 over-the-counter (OTC) service models, although both OTC models still produced generally positive results. High-end and low-end HAs showed no significant difference in outcomes.

**Meaning:**

The trial results suggest that while the lower-cost OTC service models are effective, the best outcomes were achieved with the best practice service model; support for the higher costs of high-end HAs was not identified.

## Teaser

This randomized clinical trial examines the effect of hearing aid service models and technology levels on patient outcomes for individuals with mild to moderate hearing loss.

## Introduction

Hearing loss is a public health concern, affecting about 44 million adults in the US in 2020 and projected to rise to 74 million by 2060.^1^ Untreated hearing loss affects well-being^2,3,4^ and incurs a global economic burden estimated at $980 billion.^3^ Hearing aids (HAs) can improve communication ability, quality of life, and social and emotional function^5,6^ and may potentially reduce cognitive decline.^7^ Despite these benefits, the adoption rate of HAs is low.^8,9,10^

The low uptake of HAs is influenced by several factors, including stigma and a lack of perceived need.^11,12^ Additionally, poor affordability and accessibility of HA fitting services and devices are cited as major barriers to HA adoption.^11,12,13^ Traditional fitting services, in which prescription HAs are dispensed through professionals like audiologists (referred to as the AUD service model in this article), require multiple visits for hearing tests, device customization, and maintenance. The professional services, along with the substantial patient resources needed for the fitting process (eg, transportation and time), contribute to the low affordability and accessibility of the AUD service model.

Modern HAs feature a wide range of technologies, including microphone arrays, feedback suppression algorithms, and wireless functionality. These technologies have evolved substantially over the decades, progressing from basic algorithms to more complex designs. The inclusion of high-end technologies further decreases the affordability of many HAs.

Over-the-counter (OTC) HAs have emerged as a more affordable and accessible alternative, available through pharmacies and the internet without requiring a prior clinician-patient relationship.^14^ In the OTC service model, users self-assess their hearing difficulties and self-fit/adjust devices without professional support, thereby reducing costs. To further enhance affordability, OTC HAs often incorporate lower-end technologies; however, some models (typically more expensive) are equipped with higher-end features.^15^

Can the AUD service model and high-end HA technologies deliver better patient outcomes, justifying their higher costs, compared with the OTC service model and low-end technologies? Research from randomized clinical trials (RCTs) has indicated that the OTC service model achieves comparable outcomes in domains such as communication abilities, quality of life, and speech recognition performance compared with the AUD model,^16,17^ although the OTC model tends to receive lower satisfaction ratings.^17^ Regarding HA technologies, despite commonly reported laboratory-based benefits of high-end HAs,^18^ previous clinical trials have found no statistically significant or clinically meaningful differences in communication ability, listening effort, and quality of life between high-end and low-end technologies.^18,19,20,21,22^

Taken together, current evidence suggests that low-end HAs delivered using the OTC model may yield patient outcomes comparable with high-end HAs fitted using the AUD model. However, to our knowledge, no prior research has simultaneously examined the effectiveness of HA service models and technology levels in the same study. Additionally, to our knowledge, there is no research investigating whether a hybrid service model, in which professionals fit OTC HAs (referred to as the OTC+ service model), could provide affordable and quality interventions as advocated.^23,24^ Therefore, the purpose of the present RCT was to determine the effect of HA service models (AUD, OTC+, and OTC) and technology levels (high end and low end) on patient outcomes.

## Methods

### Study Design

This 2-site RCT was conducted at the University of Iowa and Vanderbilt University Medical Center (who provided institutional review board approval) in research laboratories from February 2019 to December 2023 (Supplement 1). Participants provided written informed consent and were randomly assigned to 1 of 6 parallel groups, representing factorial combinations of 3 service models and 2 technology levels. Because OTC HAs were not defined by the US Food and Drug Administration until near the study’s completion, they were simulated using prescription HAs. A preset-based OTC HA with 4 predetermined gain-frequency responses was simulated. Patient outcomes were assessed 6 to 7 weeks post-HA fitting. The primary outcome measure was the Glasgow Hearing Aid Benefit Profile (GHABP),^25^ which was administered using ecological momentary assessment (EMA) (EMA-GHABP). EMA is a method of acquiring self-reports by repeatedly prompting respondents to report their immediate or recent clinical experiences in situ.^26^ This study followed the Consolidated Standards of Reporting Trials (CONSORT) reporting guideline.

### Participants

**Figure 1.**
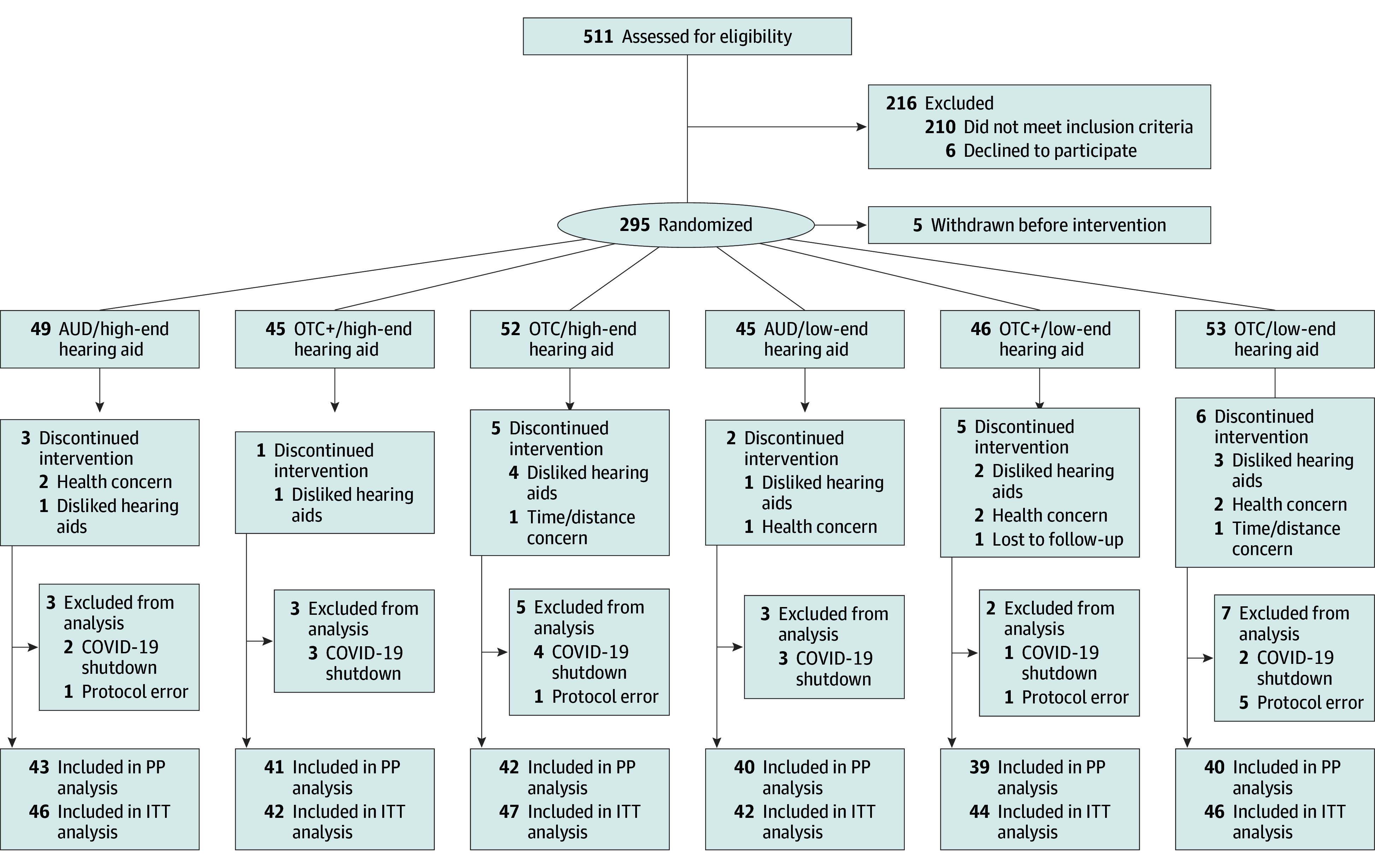
CONSORT Flowchart of Participants Through the Study AUD indicates audiologist service model; OTC, over the counter; OTC+, hybrid OTC model; PP, per-protocol analysis; ITT, intention-to-treat analysis.

Eligible participants were required to be between age 55 and 85 years; have bilateral sensorineural hearing loss with a pure-tone average at 0.5, 1, 2, and 4 kHz between 25 and 65 dB hearing loss; have no prior HA experience; and be native English speakers. Twenty-two participants withdrew due to reasons such as loss to follow-up. The participation of 23 individuals were terminated by the research team due to COVID-19 shutdowns or protocol administration errors, and they were excluded from analysis. See Figure 1 for the CONSORT reporting guideline flow diagram.

### Sample size

To test whether increasing levels of services and technology would lead to better outcomes (AUD > OTC+ > OTC; high end > low end), the trial was designed to detect a 0.3-point difference in EMA-GHABP global scores (explained later), which range from 1 to 5. This threshold was based on a previous crossover trial by Wu et al^27^ that compared patient outcomes between 2 HA devices. The results of Wu et al^27^ showed that 1 device yielded significantly higher (better) EMA-GHABP scores than the other, with a mean difference of 0.31 points, and was preferred by 29 of 39 participants (74.3%). Based on these findings, a 0.3-point difference in EMA-GHABP scores was considered a minimal important change in the present RCT, as it suggests that patients would likely prefer 1 HA intervention vs another after trying both.

Power calculations were conducted for post hoc comparisons of the main effect, specifically the pairwise comparisons between AUD, OTC+, and OTC. Given the EMA-GHABP standard deviation estimated from Wu et al,^27^ a 2-sample *t* test would achieve an 88% power to detect a difference of 0.3 points using a conservative Bonferroni adjustment to the α level of .02 (.05 of 3.0). This assumed 40 participants in each arm, totaling 80 participants in each service model for the pairwise comparisons and 240 participants in the entire study.

### Randomization

Simple randomization was used to assign participants to 1 of 6 treatment groups with equal probability based on the order of consent. The randomization sequence was generated using RANDOM.ORG.^28^

### Masking and Service Model Intervention

Masking participants to the services and HA technologies they received was not feasible. To maintain some level of blinding, participants were told that the trial had only 1 treatment group and were provided information solely on the services and technologies received. Although HA feature information was disclosed, participants were not made aware of the technology level. Research assistants responsible for collecting outcome data were masked to the treatment assignment. Services were provided by E.S. and K.B., who are licensed audiologists.

#### AUD

Audiologists fit prescription HAs following best practice guidelines.^29^ The fitting protocol included pure-tone audiometry, the Client Oriented Scale of Improvement questionnaire,^30^ measurement of loudness discomfort levels,^31,32^ and unaided QuickSIN.^33^ Audiologists configured HAs and selected slim tubes and ear domes for each participant based on assessment results, National Acoustic Laboratories second-generation prescriptions,^34^ and their clinical experiences. They also conducted probe-microphone, real-ear measures to verify HA gain and adjusted based on participant feedback. Additionally, orientation, including instructions on how and when to use HA features, and counseling were provided. Initial fitting, orientation, and counseling took a mean (SD) of 92.7 (23.6) minutes. Participants were asked to return for a follow-up appointment 1 week postfitting and were able to schedule additional follow-up visits as needed.

#### OTC+

Audiologists provided streamlined services to fit preset-based OTC HAs. This included conducting pure-tone audiometry, selecting gain-frequency response presets, and choosing ear domes. Audiologists could use the HA’s smartphone applications to adjust the devices as needed. Limited orientation and counseling were also provided. All services, except for pure-tone audiometry, were to be completed during a 30-minute session. Participants were allowed two 15-minute follow-up visits to address issues with HAs.

#### OTC

Participants took responsibility for learning to use preset-based OTC HAs without support from study audiologists. To self-select their desired presets, participants used a kiosk application on a tablet computer to listen to and compare sounds recorded from each of the 4 presets (eMethods 1, eTable 1, and eFigure 1 in Supplement 2). After choosing a preset, participants received a pair of HAs and accessories and began the trial. During the trial, participants could request a preset reselection or ask for HA replacements if they encountered device-related issues.

For all service models, participants were encouraged to use the user manual, quick start guide, and simulated HA dispenser websites created for the trial to learn about the devices. These print materials and websites mimicked the information provided by typical dispensers but used fabricated manufacturer and product names to support masking of technology level.

### Intervention: HAs

Two behind-the-ear prescription HA models (1 high end [approximately $4400 per pair] and 1 low end [approximately $1100 per pair]) were used, both from the same manufacturer and with similar appearances. See eTable 2 in Supplement 2 for the contrasts between the models. Both models were used in 2 roles: as prescription HAs (AUD) and to simulate preset-based OTC HAs (OTC+ and OTC intervention groups). The OTC HAs used a validated preset-based fitting method.^35,36^ Four presets (eTable 1 in Supplement 2), estimated to be appropriate for 67.9% of older adults with mild to moderate hearing loss,^35^ were available for selection.

### Test Measures

All measures were administered before HA fitting as baseline measures and between 6 and 7 weeks postfitting as outcome measures. The only exception was for the HA satisfaction measure, which was administered only postfitting.

#### Primary Outcome

The EMA-GHABP, a smartphone-based EMA adaptation of the GHABP,^25^ served as the primary outcome measure. The GHABP was chosen for its validated psychometric properties^25,37^ and capability to be implemented as an in situ assessment.^27^ EMA was selected for its higher responsiveness compared with retrospective self-reports.^27^

The EMA-GHABP evaluated initial hearing disability and initial hearing handicaps during the prefitting assessment session and assessed HA use, HA benefit, residual hearing disability, residual hearing handicap, and HA satisfaction during the postfitting assessment session. During each assessment session, participants completed the questionnaire on smartphones repeatedly for 1 week. The postfitting EMA-GHABP generated 2 scores: the use score, derived from items evaluating HA use, and the global score, averaged from the remaining items. See eMethods 2 and eTable 3 in Supplement 2 for more details about the EMA-GHABP.

#### Secondary Outcomes

Secondary outcome measures were implemented for comprehensive assessment. The GHABP was administered as a retrospective questionnaire (retro-GHABP), generating 2 postfitting scores: use scores and global scores. The Profile of Hearing Aid Performance (PHAP)^38^ assessed HA performance in 7 domains, with scores from the 5 domains related to speech communication averaged to derive the PHAP score. The Hearing Handicap Inventory for the Elderly (HHIE,^39^ for participants older than 65 years) or Adults (HHIA)^40^ evaluated the social and emotional effect of hearing loss. The Satisfaction with Amplification in Daily Life (SADL)^41^ assessed satisfaction with HAs.

Additionally, the Connected Speech Test (CST),^42^ a behavioral speech test, was used to measure the extent to which HAs improved speech recognition in noise. The CST was administered at +3 dB signal to noise ratio in a sound-treated booth, with speech and noise presented from 0° and 180° azimuths, respectively. The CST test condition was selected to align with those used by Humes et al,^17^ allowing for a direct comparison of results.

### Procedures

Written informed consent was obtained by Y.H.W. and T.R. Audiologists, who were masked to the treatment assignment, administered prefitting assessments in the laboratory. Participants then initiated a 1-week prefitting EMA session. Seven days later, participants returned to the laboratory where audiologists, now informed of the treatment assignment by Y.H.W. and T.R., administered the interventions. A 6-week trial then followed. At the end of the trial, participants returned to the laboratory, where research assistants, who were masked to the treatment assignment, administered outcome measures. As-worn real-ear aided responses were also measured using a probe-microphone HA analyzer. Participants then initiated a 1-week postfitting EMA session. Seven days later, participants returned to complete the remaining outcome measures and were debriefed.

### Statistical Analysis

Analyses were conducted to determine the effect of service model, technology level, and their interaction on outcomes. For the primary outcome, the EMA-GHABP use score, we dichotomized the score (ie, 1: using HAs all the time; 0: otherwise) because the data were right skewed. We used a generalized linear mixed model with a logit link function to estimate the probability that a participant would report using HAs all the time. To analyze another primary outcome, the EMA-GHABP global score, linear mixed models were used. We selected these statistical models because EMA data consisted of repeated observations within each participant. Participants completed the EMA-GHABP surveys a mean (SD) of 45.3 (12.9) times prefitting (range, 6-80) and 34.5 (13.8) times postfitting (range, 3-65). See eMethods 2 in Supplement 2 for details on EMA-GHABP data processing and analysis.

For secondary outcomes, the retro-GHABP use score was also dichotomized, and a logistic regression was used to analyze the data. Linear regression models were used for the remaining secondary outcomes.

We conducted per-protocol and intention-to-treat analyses. The per-protocol analysis included 245 participants who completed the study, while the intention-to-treat analysis included these participants along with 22 who withdrew, resulting in a total sample of 267. The intention-to-treat analysis was conducted only for the EMA-GHABP use score. For the 22 participants who withdrew, their dichotomized use score was set to 0, meaning that they would not use HAs if the outcome measures had been administered.

Study site was controlled for in all models. For the EMA-GHABP and retro-GHABP global scores, prefitting scores were controlled for in the model. For the PHAP, HHIE/HHIA, and CST, benefit scores (ie, the change in scores between before and after fitting) were used as dependent variables. Pairwise comparisons were conducted with an α level adjustment using the Tukey method. All statistical analyses were performed using R statistical software, version 4.3.0 (R Foundation).^43^ Pairwise comparisons were conducted using the emmeans package, version 1.8.6^44^ (R Foundation). For all scores reported, higher scores indicate better outcomes.

## Results

Table 1 summarizes the characteristics of participants. Of the 290 individuals who received interventions, 245 (84.5%) completed the study. Most participants were White, with 101 (41%) holding a college degree or higher. Additionally, 59 (24%) had the Montreal Cognitive Assessment^45^ scores of less than 25 points, indicating a potential for mild cognitive impairment.^46^ Mean postfitting, as-worn real-ear aided responses for a 65-dB SPL speech signal, and the number of participants requesting postfitting follow-up visits or preset reselection are shown in eFigure 2 and eTable 4 in Supplement 2, respectively.

**Table 1.** Summary Characteristics of the 245 Participants Who Completed the Study

Characteristic	No. (%)					
	High end			Low end		
	AUD (n = 43)	OTC+ (n = 41)	OTC (n = 42)	AUD (n = 40)	OTC+ (n = 39)	OTC (n = 40)
Sex						
Female	22 (51.2)	20 (48.8)	22 (52.4)	19 (47.5)	17 (43.6)	21 (52.5)
Male	21 (48.8)	21 (51.2)	20 (47.6)	21 (52.5)	22 (56.4)	19 (47.5)
Age, mean (SD), y	66.3 (7.1)	66.7 (12.4)	67.6 (7.4)	67.5 (6.0)	68.9 (7.3)	69.5 (6.8)
Race						
Asian	1 (2.3)	1 (2.4)	0	0	0	0
Black or African American	0	1 (2.4)	0	0	2 (5.1)	1 (2.5)
Multiracial^a^	1 (2.3)	1 (2.4)	1 (2.4)	0	0	2 (5.0)
White	42 (97.7)	39 (95.1)	42 (100.0)	39 (97.5)	37 (94.9)	39 (97.5)
Ethnicity						
Hispanic or Latino	2 (4.7)	2 (4.9)	0	0	0	0
Not Hispanic or Latino	41 (95.3)	39 (95.1)	41 (97.6)	40 (100.0)	39 (100.0)	40 (100.0)
Annual income >$90 000	21 (48.8)	16 (39.0)	16 (38.1)	20 (50.0)	23 (59.0)	18 (45.0)
Highest education						
Less than high school	2 (4.7)	5 (12.2)	9 (21.4)	3 (7.5)	5 (12.8)	3 (7.5)
High school	8 (18.6)	5 (12.2)	2 (4.8)	6 (15.0)	5 (12.8)	3 (7.5)
Some college	5 (11.6)	3 (7.3)	4 (9.5)	9 (22.5)	6 (15.4)	6 (15.0)
Vocational or technical degree	7 (16.3)	11 (26.8)	9 (21.4)	8 (20.0)	6 (15.4)	14 (35.0)
College degree or higher	17 (39.5)	19 (46.3)	16 (38.1)	18 (45.0)	15 (38.5)	16 (40.0)
MoCA, mean (SD)	25.9 (2.6)	26.0 (2.9)	26.2 (3.0)	25.9 (2.7)	25.5 (2.7)	25.9 (2.3)
Score <25 points	10 (23.3)	10 (24.4)	8 (19.1)	12 (30.0)	11 (28.2)	8 (20.0)
PTA						
0.5, 1, and 2 kHz, mean (SD)	31.2 (7.9)	31.8 (7.8)	30.1 (6.8)	31.5 (8.1)	30.6 (7.3)	31.0 (7.2)
0.5, 1, 2 and 4 kHz, mean (SD)	36.3 (6.6)	36.4 (6.6)	35.1 (5.9)	36.8 (7.7)	36.1 (6.1)	36.2 (5.6)

Abbreviations: AUD, audiologist service model; MoCA, Montreal Cognitive Assessment; OTC, over the counter; OTC+, hybrid OTC model; PTA, pure-tone average.

^a^ Five participants reported more than 1 race. Since each of these participants identified as White among their reported races, they are categorized as White in the article.

For all analyses conducted, the interaction between service model and technology level were not significant. Therefore, we focused the results only on the main effects.

The effects of the service model are summarized in Table 2. In the per-protocol analysis of the EMA-GHABP use score, the odds of participants reporting HA use all the time in AUD, OTC+, and OTC were estimated at 8.33 (95% CI, 5.42-12.94), 2.56 (95% CI, 1.68-3.90), and 3.35 (95% CI, 2.20-5.10), respectively. Pairwise comparisons indicated that the odds of AUD participants reporting HA use all the time were 3.25 times higher than OTC+ (95% CI, 1.60-6.69) and 2.51 times higher than OTC (95% CI, 1.22-5.10). The difference between OTC+ and OTC was not significant (odds ratio, 0.76; 95% CI, 0.38-1.55).

**Table 2.** Estimated Effect of Service Model on Outcome Scores

Variable	OR (95% CI)					
	AUD	OTC+	OTC	AUD vs OTC+^b^	AUD vs OTC^b^	OTC+ vs OTC^b^
Per-protocol analysis (n = 245)						
EMA-GHABP, use score^a^	8.33 (5.42 to 12.94)	2.56 (1.68 to 3.90)	3.35 (2.20 to 5.10)	3.25 (1.60 to 6.69)	2.51 (1.22 to 5.10)	0.76 (0.38 to 1.55)
EMA-GHABP, global score	4.16 (4.05 to 4.27)	3.83 (3.72 to 3.94)	3.85 (3.73 to 3.96)	0.33 (0.14 to 0.52)	0.32 (0.13 to 0.51)	−0.02 (−0.21 to 0.18)
Retro-GHABP, use score^a^	0.61 (0.39 to 0.97)	0.24 (0.13 to 0.42)	0.33 (0.20 to 0.56)	2.61 (1.08 to 6.23)	1.84 (0.81 to 4.18)	0.70 (0.28 to 1.77)
Retro-GHABP, global score	3.93 (3.82 to 4.05)	3.58 (3.46 to 3.69)	3.66 (3.54 to 3.78)	0.36 (0.16 to 0.55)	0.27 (0.08 to 0.47)	−0.08 (−0.28 to 0.11)
PHAP, benefit score	16.91 (13.48 to 20.35)	14.03 (10.50 to 17.55)	14.09 (10.61 to 17.56)	2.89 (−3.01 to 8.78)	2.83 (−3.03 to 8.68)	−0.06 (−5.99 to 5.87)
HHIE/HHIA, benefit score	20.49 (16.55 to 24.44)	14.16 (10.15 to 18.18)	13.87 (9.83 to 17.92)	6.33 (−0.41 to 13.07)	6.62 (−0.15 to 13.38)	0.29 (−6.54 to 7.11)
SADL	5.40 (5.20 to 5.59)	4.87 (4.67 to 5.06)	4.79 (4.59 to 4.98)	0.53 (0.20 to 0.86)	0.61 (0.28 to 0.94)	0.08 (−0.25 to 0.41)
CST, benefit score	7.55 (4.50 to 10.62)	3.73 (0.56 to 6.91)	6.97 (3.79 to 10.14)	3.82 (−1.46 to 9.10)	0.59 (−4.69 to 5.86)	−3.23 (−8.60 to 2.14)
Intention-to-treat analysis (n = 267)						
EMA-GHABP, use score^a^	6.69 (3.74 to 11.82)	1.77 (1.01 to 3.10)	1.77 (1.03 to 3.06)	3.78 (1.45 to 9.87)	3.74 (1.45 to 9.78)	0.99 (0.39 to 2.53)

Abbreviations: AUD, audiologist service model; CST, connected speech test; EMA, ecological momentary assessment; GHABP, Glasgow hearing aid benefit profile; HHIA, handicap inventory for adults; HHIE, handicap inventory for the elderly; OR, odds ratio; OTC, over the counter; OTC+, hybrid OTC model; PHAP, profile of hearing aid performance; retro, retrospective; SADL, satisfaction with amplification in daily life.

^a^ Odds ratio.

^b^ Confidence level adjustment: Tukey method for comparing a family of 3 estimates.

The per-protocol analysis results of the EMA-GHABP global score showed a similar trend. The mean score of AUD was 0.33 points higher than OTC+ (95% CI, 0.14-0.52) and 0.32 points higher than OTC (95% CI, 0.13-0.51). The difference between OTC+ and OTC was not significant (−0.02 points; 95% CI, −0.21 to 0.18).

The analysis of the retro-GHABP use and global scores generally resembled the EMA-GHABP results, showing that AUD yielded better outcomes than OTC+ and OTC, while the difference between OTC+ and OTC was not significant. However, the difference in the retro-GHABP use scores between AUD and OTC was not significant.

For the remaining secondary outcomes, the per-protocol analyses indicated that the main effects of service model were not significant, except for the SADL. AUD participants reported higher SADL scores, indicating greater HA satisfaction, compared with OTC+ by 0.53 points (95% CI, 0.20-0.86) and OTC by 0.61 points (95% CI, 0.28-0.94). The difference between OTC+ and OTC was not significant (0.08 points; 95% CI, −0.25 to 0.41).

Table 2 also presents the results of the intention-to-treat analysis on the EMA-GHABP use score. The results are identical to the per-protocol analysis, showing that AUD participants wore HAs longer than the OTC+ and OTC participants, with no significant difference observed between OTC+ and OTC.

**Figure 2.**
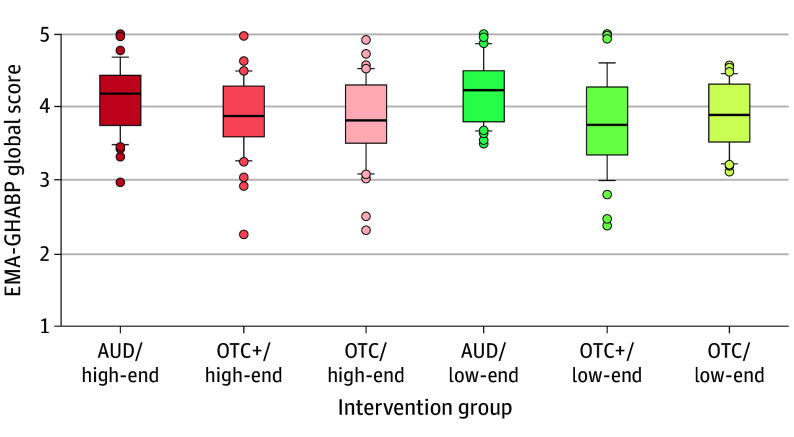
Mean Postfitting Ecological Momentary Assessment (EMA)–Glasgow Hearing Aid Benefit Profile (GHABP) Global Scores, Averaged per Participant, as a Function of Intervention Group AUD indicates audiologist service model; OTC, over the counter; OTC+, hybrid OTC model.

The effects of technology level are shown in Table 3. Across all outcome measures and in the per-protocol and intention-to-treat analyses, none of the effects were significant. To illustrate the main findings of the study, Figure 2 displays box plots of EMA-GHABP global scores as a function of intervention group. Additionally, eTable 5 in Supplement 2 summarizes the effect sizes of pairwise comparisons reported in Table 2 and Table 3.

**Table 3.** Estimated Effect of Technology Level on Outcome Scores

Variable	OR (95% CI)		
	High end	Low end	High end vs low end
Per-protocol analysis (n = 245)			
EMA-GHABP, use score^a^	3.67 (2.61 to 5.16)	4.71 (3.32 to 6.69)	0.78 (0.48 to 1.27)
EMA-GHABP, global score	3.93 (3.84 to 4.03)	3.96 (3.87 to 4.05)	−0.03 (−0.16 to 0.11)
Retro-GHABP, use score^a^	0.39 (0.26 to 0.58)	0.34 (0.22 to 0.53)	1.14 (0.63 to 2.05)
Retro-GHABP, global score	3.76 (3.66 to 3.85)	3.69 (3.59 to 3.78)	0.07 (−0.06 to 0.21)
PHAP, benefit score	15.71 (12.91 to 18.51)	14.31 (11.43 to 17.19)	1.40 (−2.62 to 5.42)
HHIE/HHIA, benefit score	17.25 (14.01 to 20.49)	15.10 (11.81 to 18.39)	2.15 (−2.47 to 6.77)
SADL	5.07 (4.92 to 5.23)	4.96 (4.80 to 5.12)	0.11 (−0.11 to 0.34)
CST, benefit score	7.58 (5.04 to 10.12)	4.59 (2.01 to 7.17)	2.99 (−0.63 to 6.61)
Intention-to-treat analysis (n = 267)			
EMA-GHABP, use score^a^	2.66 (1.70 to 4.22)	2.83 (1.79 to 4.48)	0.94 (0.49 to 1.80)

Abbreviations: CST, connected speech test; EMA, ecological momentary assessment; GHABP, Glasgow hearing aid benefit profile; HHIA, handicap inventory for adults; HHIE, handicap inventory for the elderly; OR, odds ratio; PHAP, profile of hearing aid performance; retro, retrospective; SADL, satisfaction with amplification in daily life.

^a^ Odds ratio.

## Discussion

### Effect of Service

Our study results aligned with previous research in several ways. Consistent with the findings from the RCTs by Humes et al^17^ and De Souza et al,^16^ we found no service model effect on speech communication (PHAP), hearing handicap (HHIE/HHIA), and laboratory-based speech recognition performance (CST). As Humes et al^17^ observed, the participants in our trial reported higher satisfaction with AUD compared with OTC (SADL).

However, our study introduced new insights with the EMA-GHAPB that showed that AUD outperformed OTC. The observed EMA-GHABP global score differences (0.32 points; Table 2) exceeded the minimal important difference (0.3 points), indicating that patients would likely prefer audiologist-fitted HAs vs OTC HAs if given the opportunity to try both.

The differences between our findings and those of Humes et al and De Souza et al can be attributed to several factors. First, the outcome measurement tools differed: we used EMA to collect in-situ self-reports, while Humes et al^17^ and De Souza et al^16^ used retrospective questionnaires. The in-situ nature of EMA (which reduces memory bias) and repeated sampling (which provides more data points) make it more responsive than retrospective questionnaires in capturing HA outcome differences.^27^ Second, the participant inclusion criteria differed. Our study, like the one from Humes et al,^17^ included only new HA users, whereas De Souza et al^16^ included new and experienced users. Experienced users may have had the knowledge and skills necessary to successfully use OTC HAs, which could lead to better outcomes. Third, the fitting methods of OTC HAs differed: we used preset-based HAs, whereas De Souza et al^16^ used self-fitting HAs that determine fitting parameters based on hearing thresholds measured by the device. Although research directly comparing these 2 fitting methods is currently unavailable, self-fitting HAs may produce frequency responses more closely tailored to an individual’s specific hearing loss, potentially yielding better outcomes compared with preset-based HAs.

The finding that AUD outperformed OTC raises an important question: was the outcome difference driven by the service itself, the participant’s personal interactions with audiologists, or both? While our RCT could not directly address this, evidence suggests that the outcome difference was primarily driven by the service itself. A recent study demonstrated that, when HA configurations were held constant, an HA fitting process involving extensive audiologist-patient interactions did not yield better outcomes compared with a fitting process with minimal interactions.^47^

We found no evidence suggesting that OTC+ was superior to OTC, indicating that the limited services provided in OTC+ did not enhance outcomes. The study audiologists observed that when OTC+ participants faced device-related issues, such as acoustic feedback, the available solutions (eg, switching presets) were often insufficient to resolve the problems. Optimal outcomes may require more in-depth services, including probe-microphone measures, and greater flexibility in configuring devices using fitting software.

Although OTC+ and OTC had poorer outcomes compared with AUD, their mean EMA-GHABP global scores were close to 4 points (Table 2 and Figure 2), reflecting generally positive outcomes (eg, very satisfied on the HA satisfaction question of the GHABP). Therefore, OTC+ and OTC are considered effective service models.

### Effect of Technology

Consistent with prior clinical trials,^18,20,21,22^ we did not find that high-end HAs provided better outcomes than low-end HAs. This finding suggests that for the same generation of devices, older adults with mild to moderate hearing loss are unlikely to perceive additional general benefits from high-end HAs compared with low-end HAs in their daily lives. However, it is essential to clarify that many current low-end HAs incorporate technologies that were considered advanced in earlier generations, and our study did not compare devices across different generations. Therefore, our results should not be interpreted as implying that HA technologies do not improve patient outcomes.

### Limitations

This study had several limitations. First, it used only 1 preset-based OTC device by simulation, which may not have captured the variability and range of OTC HAs available on the market. Second, because participants were randomly assigned to intervention groups, the OTC participants in our study may not represent clinical OTC HA users, who tend to be younger and report milder hearing difficulties compared with prescription HA users.^48,49^ These limitations could have affected the generalizability of the study’s findings. Third, the study was not powered to detect interaction effects between service and technology; thus, any interaction analyses should be interpreted as exploratory.

## Conclusions

This RCT found that the OTC+ and OTC service models were effective but did not achieve the same outcomes as the AUD service model. The limited services offered by the OTC+ model did not enhance outcomes compared with the OTC model. For the same generation of HAs, high-end and low-end devices yielded similar general patient-reported outcomes in clinical settings.

## Notes

Supplement 1. Trial protocol

Supplement 2. eMethods 1. Preset-Based Over-the-Counter Hearing Aids eTable 1. Gain-Frequency Responses of Four Presets eFigure 1. Screenshots of the Hearing Aid Selector Kiosk App eTable 2. Differences, as Described by the Manufacturer, Between High-End and Low-End Hearing Aids eMethods 2. Glasgow Hearing Aid Benefit Profile (GHABP) as an Ecological Momentary Assessment (EMA) Survey: Implementation, Processing, and Analysis eTable 3. EMA-GHABP Questions and Response Options eFigure 2. Mean Post-Fitting, As-Worn Real-Ear Aided Response for a 65 dB SPL Speech Input Level eTable 4. Number of Participants Requesting Follow-Up Laboratory Visits eTable 5. Effect Sizes of Pairwise Comparisons eReferences.

Supplement 3. Data sharing statement
