# Supplementary material for: Hearing Aid Service Models, Technology, and Patient Outcomes: A Randomized Clinical Trial
Source: JAMA Otolaryngol Head Neck Surg. 2025 May 15;151(7):684–92. doi: 10.1001/jamaoto.2025.1008 (PMC12082484; doi:10.1001/jamaoto.2025.1008)
Supplement: Supplement 1. — Trial protocol [file jamaotolaryngolheadnecksurg-e251008-s001.pdf]

Impact of hearing aid service-delivery model and technology on patient outcomes: A Randomized Clinical Trial

Trial Protocol and Statistical Analysis Plan

Version: Final

1 ADMINISTRATIVE INFORMATION

1.1 Trial registration

ClinicalTrials.gov Identifier: NCT03579563

1.2 Funding

The study was funded by NIH/NIDCD R01DC015997.

1.3 Roles and responsibilities

Name	Contact information	Affiliations	Role and responsibilities
Wu, Yu-Hsiang	yu-hsiang-wu@uiowa.edu	Department of Communication Sciences and Disorders, The University of Iowa	Principal investigator: funding acquisition, study design conceptualization, project supervision, report writing
Ricketts, Todd	todd.a.ricketts@vumc.org	Department of Hearing & Speech Sciences, Vanderbilt University Medical Center	Principal investigator: funding acquisition, study design conceptualization, project supervision, report writing
Oleson, Jacob	jacob-oleson@uiowa.edu	Department of Biostatistics, The University of Iowa	Biostatistician: data processing, data analysis, report writing
Stangl, Elizabath	elizabeth-stangl@uiowa.edu	Department of Communication Sciences and Disorders, The University of Iowa	Audiologist: hearing aid fitting, data collection, data curation, report writing
Branscome, Kjersten	kjersten.branscome@vumc.org	Department of Hearing & Speech Sciences, Vanderbilt University Medical Center	Audiologist: hearing aid fitting, data collection, data curation

2 INTRODUCTION

2.1 Background and rationale

Age-related hearing loss presents a significant national challenge due to its high prevalence and significant quality-of-life consequences. Although hearing aids (HAs) are the primary choice for managing age-related hearing loss, their adoption rate remains low. One commonly reported reason for not adopting HAs is the financial and physical barriers associated with traditional hearing healthcare, which involves multiple visits to licensed professionals (such as audiologists) for diagnosis and a lengthy process of fitting and fine-tuning prescription HAs. This traditional service-delivery model is referred to as the *AUD service model*.

The high cost of HA devices further exacerbates the accessibility and affordability challenges of traditional hearing healthcare. Specifically, a variety of technologies and features have been implemented in modern HAs, including multi-channel wide dynamic range compression, directional microphones, noise reduction algorithms, and wireless functionality. These technologies have evolved from basic algorithms into more sophisticated and complex designs. However, these high-end technologies and features often make HAs more expensive and less affordable.

In recent years, over-the-counter (OTC) HAs have emerged as an alternative to address some of the affordability and accessibility issues of the AUD service. This direct-to-consumer model enables users to self-determine hearing loss, self-fit HAs, and self-manage the device without the need for

professional support. This direct-to-consumer model is referred to as the *OTC service model*. Additionally, it has been advocated that a hybrid service model, where professionals fit OTC HAs (referred to as the *OTC+ service model*), could offer affordable and quality amplification interventions.

Previous clinical trials have shown that the OTC service model yields outcomes comparable to the AUD service model. Furthermore, prior research has found no statistically significant or clinically important differences in patient outcomes between high-end and low-end HAs. However, no prior research has systematically examined the effectiveness of HA fitting service models and HA technology levels in the same study. Additionally, no prior research has investigated the patient outcomes of the OTC+ service model.

2.2 Objective

To determine the effect of HA fitting services (AUD, OTC+, and OTC) and technology levels (high-end and low-end) on patient outcomes.

2.3 Trial design

This is a two-site randomized controlled trial. Participants will be randomly assigned to one of six parallel arms, which are factorial combinations of three service models (AUD, OTC+, and OTC) and two HA technology levels (high-end and low-end). Baseline measures will be administered before HA fitting and patient outcomes will be assessed six to seven weeks post-HA fitting.

3 METHODS: PARTICIPANTS, INTERVENTIONS, AND OUTCOMES

3.1 Study site

The study will be conducted in the Hearing Aid and Aging Research Laboratory, Department of Communication Sciences and Disorders, the University of Iowa and the Dan Maddox Hearing Aid Research Laboratory, Department of Hearing & Speech Sciences, the Vanderbilt University Medical Center. The two sites, one in Iowa City (a more rural locale) and the other in Nashville (a more urban locale), provide us with the opportunity to engage a diverse range of participants.

3.2 Eligibility criteria

3.2.1 Inclusion criteria:

- Adults between 55 and 85 years old
- Adult-onset, bilateral, mild-to-moderate sensorineural hearing loss
    - Pure-tone average across 500, 1000, 2000, and 4000 Hz between 25 and 55 dB HL
    - Thresholds from 500-4000 Hz no poorer than 65 dB HL, with up to 2 thresholds outside this criterion by < 10 dB still being eligible.

3.2.2 Exclusion criteria:

- Non-native speaker of English
- Had prior HA experience

3.3 Arms

- <u>AUD/High-end</u>: In this group, audiologists will fit bilateral high-end prescription HAs according to the best practice guidelines.
- <u>OTC+/High-end</u>: In this group, audiologists will provide brief services to fit bilateral preset-based OTC-HAs, which are simulated using high-end prescription HAs.

- OTC/High-end: In this group, a pair of preset-based OTC HAs, simulated using high-end prescription HAs, will be provided to each subject. Participants will take the full initiative and responsibility for learning and using the HAs without assistance from audiologists.
- AUD/Low-end: In this group, audiologists will fit bilateral low-end prescription HAs according to the best practice guidelines.
- OTC+/Low-end: In this group, audiologists will provide brief services to fit bilateral preset-based OTC-HAs, which are simulated using low-end prescription HAs.
- OTC/Low-end: In this group, a pair of preset-based OTC HAs, simulated using low-end prescription HAs, will be provided to each subject. Participants will take the full initiative and responsibility for learning and using the HAs without assistance from audiologists.

3.3.1 Interventions: HA fitting services

The services will be provided by audiologists in the Hearing Aid and Aging Research Laboratory, Department of Communication Sciences and Disorders, the University of Iowa and the Dan Maddox Hearing Aid Research Laboratory, Department of Hearing & Speech Sciences, the Vanderbilt University Medical Center.

3.3.1.1 AUD service model

In this service model, audiologists will fit prescription HAs according to the best practice guidelines. The fitting protocol will include the following measures: pure-tone audiometry to determine hearing threshold, the Client Oriented Scale of Improvement questionnaire (COSI)[1] to evaluate individual listening needs, measurement of loudness discomfort levels (LDL)[2,3] to determine sound levels that are uncomfortably loud, and the QuickSIN[4] to assess unaided speech recognition performance in noise. Audiologists will configure the HAs and select the appropriate slim tube length, ear dome size, and type for each participant based on assessment results, NAL-NL2 prescriptions,[5] and their clinical experiences. Audiologists will then conduct probe-microphone real-ear measures to verify the gains of the HAs, and make further adjustments based on participant's feedback for comfort and sound quality. Additional hearing aid memories will be added and features will be adjusted based on the participants' listening needs. Audiologists will also provide orientation (e.g., insertion, removal, and care of devices, smartphone app installation and orientation) and counseling (e.g., establishing realistic expectations of amplification) services. Participants were asked to return for a follow-up appointment one-week post-fitting and were able to schedule additional follow-up visits as needed.

3.3.1.2 OTC+ service model

In this service model, audiologists will provide services to fit preset-based OTC HAs. These services include conducting pure-tone audiometry to determine hearing thresholds and selecting the appropriate preset (from the four available presets, detailed below), slim tube length, ear dome size, and type. Selections will be based on the participant's audiogram, NAL-NL2 targets, and the audiologist's clinical experience. Audiologists can use the volume control and smartphone apps to make adjustments to the HAs as needed, but they will not use HA fitting software to program the devices as prescription HAs. Audiologists will also provide brief HA orientation and counseling. All services, except for pure-tone audiometry, will be completed in a 30-minute session. Participants will be allowed up to two follow-up visits of 15 minutes each for additional instruction or to address issues with the devices.

3.3.1.3 OTC service model

In this service model, participants will self-fit preset-based OTC HAs. To self-select the OTC HA's preset, participants will use a Hearing Aid Selector kiosk app installed on a tablet computer to listen

to sounds recorded from each preset. The selection process will be conducted without assistance from audiologists. After selecting the preferred preset for each ear, participants will receive a package containing a pair of OTC HAs, which are simulated using prescription HAs (detailed below), and accessories including domes, slim tubes, and cleaning tools. The HAs will come with medium-length slim tubes and medium-sized dome tips of the style defaulted to the selected preset. The package will also include slim tubes of different lengths and various dome types in different sizes for replacement. During the trial period, participants will be permitted to return to the laboratory to reselect presets or request HA replacements if they encounter device-related issues they cannot resolve.

3.3.2 Interventions: Hearing aids

Two commercially available behind-the-ear prescription HA models will be used: a high-end model (retail price per pair ≈ $4,400 at the commencement of the study in 2018) and a low-end model (retail price per pair ≈ $1,100). Both models are from the same manufacturer but have different chipsets. HAs are coupled to the user's ears using slim tubes and non-custom dome tips, Participants will not be able to recognize the HA model based on the device case or the smartphone apps. The contrasts between high-end and low-end HAs are shown in the table below.

Feature	High-end HA	Low-end HA
Signal processing channels	48	12
Hearing programs	6	4
Extended dynamic range	Yes	No
Extended bandwidth	Yes	No
Directionality	Automatic/Adaptive	Automatic/Fixed
Narrow directionality	Yes	No
General-purpose noise reduction	Yes	Yes
Impulse noise reduction	Yes	No
Spatial noise reduction	Yes	No
Reverberation reduction	Yes	No
Wind noise reduction	Yes	No
Feedback cancellation	Yes	Yes
Smartphone app	Yes	Yes
Volume control	Yes	Yes
Program control	Yes	Yes
Gain-frequency response adjustment	Yes	No
Directionality control (direction and width of the beam)	Yes	No

3.3.2.1 OTC HAs simulation

OTC HAs will be simulated using prescription HAs. OTC HAs will use a validated preset-based fitting method developed from audiometric data sourced from the National Health and Nutrition Examination Survey (NHANES).[6,7] Four presets (i.e., four pre-determined gain-frequency responses) are available for selection. It is estimated that these four presets are appropriate for 67.9% of the NHANES older adults with mild-to-moderate hearing loss. Both high-end and low-end HAs will be used to simulate OTC HAs.

3.3.3 Materials used in interventions

All participants will be encouraged to use the user manual, quick start guide, and simulated HA dispenser websites created for the trial to learn about the devices. These websites offer information about hearing loss, as well as the specifications, use, care, and troubleshooting of HAs through text and videos. They also include a frequently asked questions page. These print materials and websites mimic the information provided by typical dispenser websites but use fabricated manufacturer and product names. The simulated websites for the high-end and low-end HAs are https://www.precisehearingcare.com/ and https://www.exacthearingcare.com/, respectively.

3.4 Outcomes

3.4.1 Primary outcomes

3.4.1.1 In-situ Glasgow Hearing Aid Benefit Profile: HA use (EMA-GHABP, Use score)

The GHABP[8] is a questionnaire that measures HA users' listening experience in four situations: TV listening, small conversation in quiet, conversation in noise, and group conversation. The GHABP will be administered using smartphone-based Ecological Momentary Assessment (EMA) to collect in-situ self-reports. The GHABP items evaluate HA use averaged across the four listening situations form the EMA-GHABP Use score. The score ranges from 1 (not use HA at all) to 5 (use HA all the time). The EMA-GHABP Use score will be measured between 6- and 7-weeks post-intervention for one week.

3.4.1.2 In-situ Glasgow Hearing Aid Benefit Profile: Global score (EMA-GHABP, Global score)

The EMA-GHABP Global score is derived from the items of the in-situ GHABP that evaluate a patient's hearing disability, hearing handicap, HA benefit, and HA satisfaction across the four listening situations. This score ranges from 1 (poorer outcome) to 5 (better outcome). It will be measured for one week both pre-intervention and between 6 and 7 weeks post-intervention. During the pre-intervention assessment, only the items related to hearing disability and hearing handicap will be used.

3.4.2 Secondary outcomes

3.4.2.1 Retrospective Glasgow Hearing Aid Benefit Profile: HA use (Retro-GHABP, Use score)

The GHABP will also be administered as a retrospective questionnaire. The items evaluate patient's HA use averaged across the four listening situations derive the Retro-GHABP Use score. The score ranges from 1 (not use HA at all) to 5 (use HA all the time). The Retro-GHABP Use score will be measured at 6-week post intervention.

3.4.2.2 Retrospective Glasgow Hearing Aid Benefit Profile: Global score (Retro-GHABP, Global score)

The Retro-GHABP Global score is derived from the items of the retrospective GHABP that evaluate a patient's hearing disability, hearing handicap, HA benefit, and HA satisfaction across the four listening situations. The score ranges from 1 (poorer outcome) to 5 (better outcome). The Retro-GHABP Global score will be measured pre-intervention and at 6 weeks post-intervention. In the pre-intervention assessment, only the items related to hearing disability and hearing handicap will be used.

3.4.2.3 Profile of Hearing Aid Performance (PHAP)

The PHAP[9] is a questionnaire designed to measure the performance of HAs across various listening situations in seven domains. The scores of the five domains related to speech communication will be averaged to derive the PHAP score. The score ranges from 1 (good performance) to 99 (poor performance). Participants will complete this questionnaire pre-intervention and at 7 weeks post-

intervention. The difference between pre- and post-intervention scores (benefit score; referred to as PHAP-Benefit) will be used in the analysis. PHAP-Benefit ranges from -99 (intervention is detrimental) to 99 (intervention is beneficial).

3.4.2.4 Hearing Handicap Inventory for the Elderly (HHIE) or Hearing Handicap Inventory for Adults (HHIA)

The HHIE[10] and HHIA[11] are questionnaires designed to measure participant's perceived hearing handicap. For participants aged 65 and over and younger than 65 years old,, the HHIE and HHIA will be used, respectively. The score ranges from 0 (no handicap) to 100 (more handicap). Participants will complete this questionnaire pre-intervention and at 7 weeks post-intervention. The difference between pre- and post-intervention scores (benefit score; referred to as HHIE/A-Benefit) will be used in the analysis. HHIE/A-Benefit ranges from -100 (intervention is detrimental) to 100 (intervention is beneficial).

3.4.2.5 Satisfaction With Amplification in Daily Life (SADL)

The SADL[12] is a questionnaire designed to measures participant's perceived HA satisfaction. The score ranges from 1 (low satisfaction) to 7 (high satisfaction). Participants will complete this questionnaire at 7 weeks post-intervention.

3.4.2.6 Connected Speech Test (CST)

The CST[13] is a speech recognition test designed to simulate daily speech communication. The CST will be administered in a sound-treated booth. Speech and noise will be presented at 0 and 180 degrees azimuth at a +3 dB signal-to-noise ratio (speech = 65 dBA). The score ranges from 0 (understand no speech) to 100 (understand all speech). Participants will complete this test pre-intervention and at 6 weeks post-intervention. The difference between pre- and post-intervention scores (benefit score; referred to as CST-Benefit) will be used in the analysis. CST-Benefit ranges from --100 (intervention is detrimental) to 100 (intervention is beneficial).

3.5 Sample size

The trial is designed to detect a 0.3-point difference in the primary outcome GHABP. This difference is considered a minimal important change (MIC), as participants would prefer one HA intervention over another if they had the opportunity to try both.[14] Power calculations are conducted for post-hoc comparisons of the main effects, specifically the pairwise comparisons between AUD, OTC+, and OTC. Based on the MIC and an estimated standard deviation from prior research (0.53 points), a two-sample t-test would achieve 88% power to detect a difference of 0.3 points, using a conservative Bonferroni adjustment to the alpha level of 0.0167 (0.05/3), assuming 40 participants in each of the six arms, totaling 80 participants in each service model for the pairwise comparisons. Therefore, a total of 240 participants will complete the trial, with 120 participants in each study site.

3.6 Allocation

The treatment allocation sequence is generated using computer-generated random numbers. The assignment of treatment is based on the order of being eligible to participate in the study.

3.7 Blinding

Participants will be kept blind to the HA technology level. However, blinding participants to the services they receive is not feasible. Therefore, the study will employ a form of deception where participants will be informed that the trial has only one treatment arm and will be provided information solely on the services they receive. The research assistants responsible for collecting outcome data will remain blinded to the treatment assignment.

3.8 Participant timeline

Participants for this clinical trial will be recruited through print, radio, and online advertisements, as well as mass emails/letters to established registries/lists. The principal investigators of the trial will obtain consent from participants. Following enrollment, audiologists who are blinded to the treatment assignment will administer pre-intervention assessments. Participants will then start the one-week EMA-GHABP pre-intervention measure. Seven days later, participants will return to the laboratory where audiologists, now informed of the treatment assignment, will administer the interventions. A 6-week trial will then follow. At the end of the trial, participants will return to the laboratory where research assistants, who are blinded to the treatment assignment, will administer outcome measures. Participants will then start the one-week EMA-GHABP post-intervention measure. Seven days later, participants will return to the laboratory to complete the remaining outcome measures. Participants will be debriefed and compensated for their participation. Refer to Figure 1 for the participant timeline.

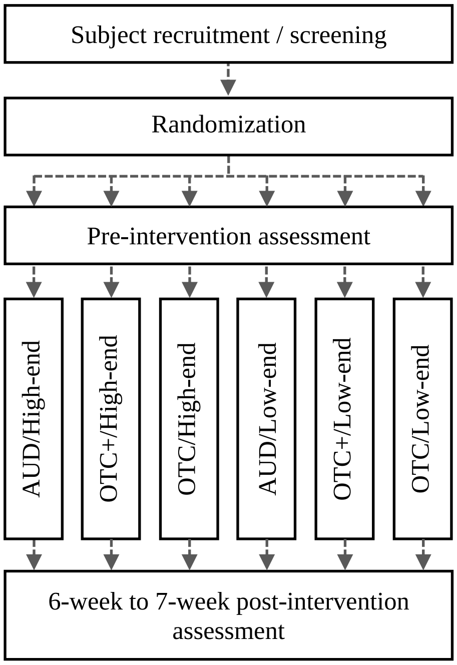

Figure 1. *Participant timeline*

3.9 Adverse events

Adverse events will be monitored for each subject during their participation in the study, which average 8 weeks from the time the participants entered the study.

4 STATISTICAL ANALYSIS PLAN

Analyses will be conducted to determine the effect of service model, technology level, and their interaction on outcomes. For the primary outcomes, the EMA-GHABP Use score and EMA-GHABP Global score, linear mixed models (LMM) will be used. LMM is selected because the primary outcomes will be collected using the EMA methodology and therefore consists of repeated observations within each participant. Linear regression models will be used for the secondary outcome dependent variables, including Retro-GHABP Use score, Retro-GHABP Global score, PHAP-Benefit, HHIE/A-Benefit, SADL, and CST-Benefit scores.

For all analyses, study site (Iowa and Tennessee) will be controlled for in the model. For both EMA-GHABP Global score and Retro-GHABP Global score, the pre-intervention GHABP scores will be controlled for in the model, with the repeated observations of pre-intervention EMA-GHABP Global score being averaged within each participant before being controlled for in the LMM. Pairwise comparisons (e.g., AUD vs. OTC+) will be conducted with an alpha level adjustment using the Tukey method. Two-tailed tests will be employed, with a significance level of .05. All statistical analyses will be performed using R statistical software.

4.1 Missing data

There will likely be participants who cannot be reached during the follow-up or decide to withdraw from the study. We plan to conduct both per-protocol (PP) analysis on participants who completed the study and intention-to-treat (ITT) analysis on all enrolled participants. The ITT analysis will be conducted only for the primary outcome, the EMA-GHABP Use score, which represents HA use. For those who withdraw from the study, their EMA-GHABP Use scores will be set to 0, indicating they would not use HAs if the outcome measures had been administered.

REFERENCES

1. Dillon H, James A, Ginis J. Client Oriented Scale of Improvement (COSI) and its relationship to several other measures of benefit and satisfaction provided by hearing aids. *Journal of the American Academy of Audiology*. 1997;8(1):27-43.
2. Cox RM. Using loudness data for hearing aid selection: The IHAFF approach. *The Hearing Journal*. 1995;48(2):10-39.
3. Ricketts TA, Bentler R, Mueller HG. Pre-Fitting Tests Using Frequency-Specific Measures. *Essentials of modern hearing aids: Selection, fitting, and verification*. Plural Publishing; 2017:101-133.
4. Killion MC, Niquette PA, Gudmundsen GI, Revit LJ, Banerjee S. Development of a quick speech-in-noise test for measuring signal-to-noise ratio loss in normal-hearing and hearing-impaired listeners. *The Journal of the Acoustical Society of America*. 2004;116(4):2395-2405.
5. Keidser G, Dillon H, Flax M, Ching T, Brewer S. The NAL-NL2 prescription procedure. *Audiology Research*. 2011;1(1):e24.
6. Urbanski D, Hernandez H, Oleson J, Wu Y-H. Toward a new evidence-based fitting paradigm for over-the-counter hearing aids. *American Journal of Audiology*. 2021;30(1):43-66.
7. Venkitakrishnan S, Urbanski D, Wu Y-H. Efficacy and Effectiveness of Evidence-Based Non–Self-Fitting Presets Compared to Prescription Hearing Aid Fittings and a Personal Sound Amplification Product. *American Journal of Audiology*. 2024;33(1):31-54.
8. Gatehouse S. Glasgow Hearing Aid Benefit Profile: Derivation and validation of a client-centered outcome measure for hearing aid services. *Journal of the American Academy of Audiology*. 1999;10(02):80-103.
9. Cox RM, Gilmore C. Development of the profile of hearing aid performance (PHAP). *Journal of Speech, Language, and Hearing Research*. 1990;33(2):343-357.
10. Ventry I, Weinstein B. The hearing handicap inventory for the elderly: a new tool. *Ear and Hearing*. 1982;3(3):128-134.
11. Newman CW, Weinstein BE, Jacobson GP, Hug GA. The Hearing Handicap Inventory for Adults: psychometric adequacy and audiometric correlates. *Ear and Hearing*. 1990;11(6):430-433.
12. Cox RM, Alexander GC. Measuring satisfaction with amplification in daily life: The SADL scale. *Ear and Hearing*. 1999;20(4):306-320.
13. Cox RM, Alexander GC, Gilmore C. Development of the connected speech test (CST). *Ear and Hearing*. 1987;8(5):119S-126S.
14. Wu Y-H, Stangl E, Chipara O, Gudjonsdottir A, Oleson J, Bentler R. Comparison of in-situ and retrospective self-reports on assessing hearing aid outcomes. *Journal of the American Academy of Audiology*. 2020;31(10):746-762.
