# Supplementary material for: Hearing Aid Service Models, Technology, and Patient Outcomes: A Randomized Clinical Trial
Source: JAMA Otolaryngol Head Neck Surg. 2025 May 15;151(7):684–92. doi: 10.1001/jamaoto.2025.1008 (PMC12082484; doi:10.1001/jamaoto.2025.1008)
Supplement: Supplement 2. — eMethods 1. Preset-Based Over-the-Counter Hearing Aids eTable 1. Gain-Frequency Responses of Four Presets eFigure 1. Screenshots of the Hearing Aid Selector Kiosk App eTable 2. Differences, as Described by the Manufacturer, Between High-End and Low-End Hearing Aids eMethods 2. Glasgow Hearing Aid Benefit Profile (GHABP) as an Ecological Momentary Assessment (EMA) Survey: Implementation, Processing, and Analysis eTable 3. EMA-GHABP Questions and Response Options eFigure 2. Mean Post-Fitting, As-Worn Real-Ear Aided Response for a 65 dB SPL Speech Input Level eTable 4. Number of Participants Requesting Follow-Up Laboratory Visits eTable 5. Effect Sizes of Pairwise Comparisons eReferences. [file jamaotolaryngolheadnecksurg-e251008-s002.pdf]

Supplemental Online Content

Wu Y-H, Stangl E, Branscome K, Oleson J, Ricketts T. Hearing aid service models and technology and patient outcomes: a randomized clinical trial. *JAMA Otolaryngol Head Neck Surg*. Published online May 15, 2025. doi:10.1001/jamaoto.2025.1008

eMethods 1. Preset-Based Over-the-Counter Hearing Aids

eTable 1. Gain-Frequency Responses of Four Presets

eFigure 1. Screenshots of the Hearing Aid Selector Kiosk App

eTable 2. Differences, as Described by the Manufacturer, Between High-End and Low-End Hearing Aids

eMethods 2. Glasgow Hearing Aid Benefit Profile (GHABP) as an Ecological Momentary Assessment (EMA) Survey: Implementation, Processing, and Analysis

eTable 3. EMA-GHABP Questions and Response Options

eFigure 2. Mean Post-Fitting, As-Worn Real-Ear Aided Response for a 65 dB SPL Speech Input Level

eTable 4. Number of Participants Requesting Follow-Up Laboratory Visits

eTable 5. Effect Sizes of Pairwise Comparisons

eReferences.

This supplemental material has been provided by the authors to give readers additional information about their work.

© 2025 Wu Y-H et al. *JAMA Otolaryngology–Head & Neck Surgery*.

eMethods 1. Preset-Based Over-the-Counter Hearing Aids

Presets

In this study, over-the-counter (OTC) hearing aids (HAs) were simulated using prescription HAs. OTC HAs employed a validated preset-based fitting method developed from audiometric data sourced from the National Health and Nutrition Examination Survey.[1,2] Preset-based OTC HAs were chosen over self-fit OTC HAs because they do not require mobile devices for configuration, potentially making them more affordable and accessible. In addition, preset-based HAs are, as of this writing in 2024, commonly available in the OTC marketplace representing approximately 80% of FDA-approved devices.[3] Four presets, each with a pre-determined gain-frequency response, were available for selection (see **eTable 1** below). The default ear domes for Presets 1 and 2 are open domes, while Presets 3 and 4 use closed domes by default.

Preset selection for the OTC service model

To assist participants in selecting presets in the OTC/High-end and OTC/Low-end intervention groups, two Hearing Aid Selector kiosk apps—one for each technology level—were developed for tablet computers. These apps allowed participants to listen to and compare sounds recorded from each of the four presets.

To record the sounds, each of the four presets were programmed to a pair of HAs used in the study. The HAs were coupled to the ears of the Knowles Electronics Manikin for Acoustic Research (KEMAR) using the default ear dome of each preset. Recordings were made of the HAs' output for each preset and volume control step using speech in quiet (60 dBA), speech in an 8-talker babble noise (speech=67.4 dBA, noise=63.7 dBA), and music (a 73-second clip of "At Last" by Etta James, at a level comfortable for people with normal hearing) in a sound-treated booth.

The recorded sounds were presented by the kiosk app to participants using TDH-50P headphones. Inverse filters were applied to the recordings to ensure that the headphones and playback system were acoustically transparent.

During preset selection, participants were seated in the sound-treated booth and instructed to imagine they were selecting their HAs in a self-directed process, similar to choosing reading glasses at a retail or drug store. The kiosk app guided them on how to navigate the process (see **eFigure 1** below for examples), with printed instructions also provided. Participants were asked to refer to the kiosk app's Frequently Asked Questions section if they had questions. They were also advised to switch between different presets and recordings (i.e., speech in quiet, speech in noise, and music), adjust the volume within each of the four presets to simulate HA's volume control, and take as much time as needed to listen. To maintain the self-selection aspect of the OTC service model, participants were told that the study audiologist was not allowed to answer questions or guide them through the selection process.

© 2025 Wu Y-H et al. *JAMA Otolaryngology–Head & Neck Surgery*.

eTable 1. Gain-Frequency Responses of Four presets.

	Real-ear aided response (dB SPL) for a 65 dB SPL speech input level				
	250 Hz	500 Hz	1 kHz	2 kHz	4 kHz
Preset 1	56	58	56	70	72
Preset 2	57	61	58	67	65
Preset 3	59	60	61	75	76
Preset 4	56	65	63	73	74

© 2025 Wu Y-H et al. *JAMA Otolaryngology–Head & Neck Surgery*.

eFigure 1. Screenshots of the Hearing Aid Selector Kiosk App. The four presets are labeled as EP, LP, SP, and XP in the app. The term *Precise* shown in the app refers to the high-end hearing aids.

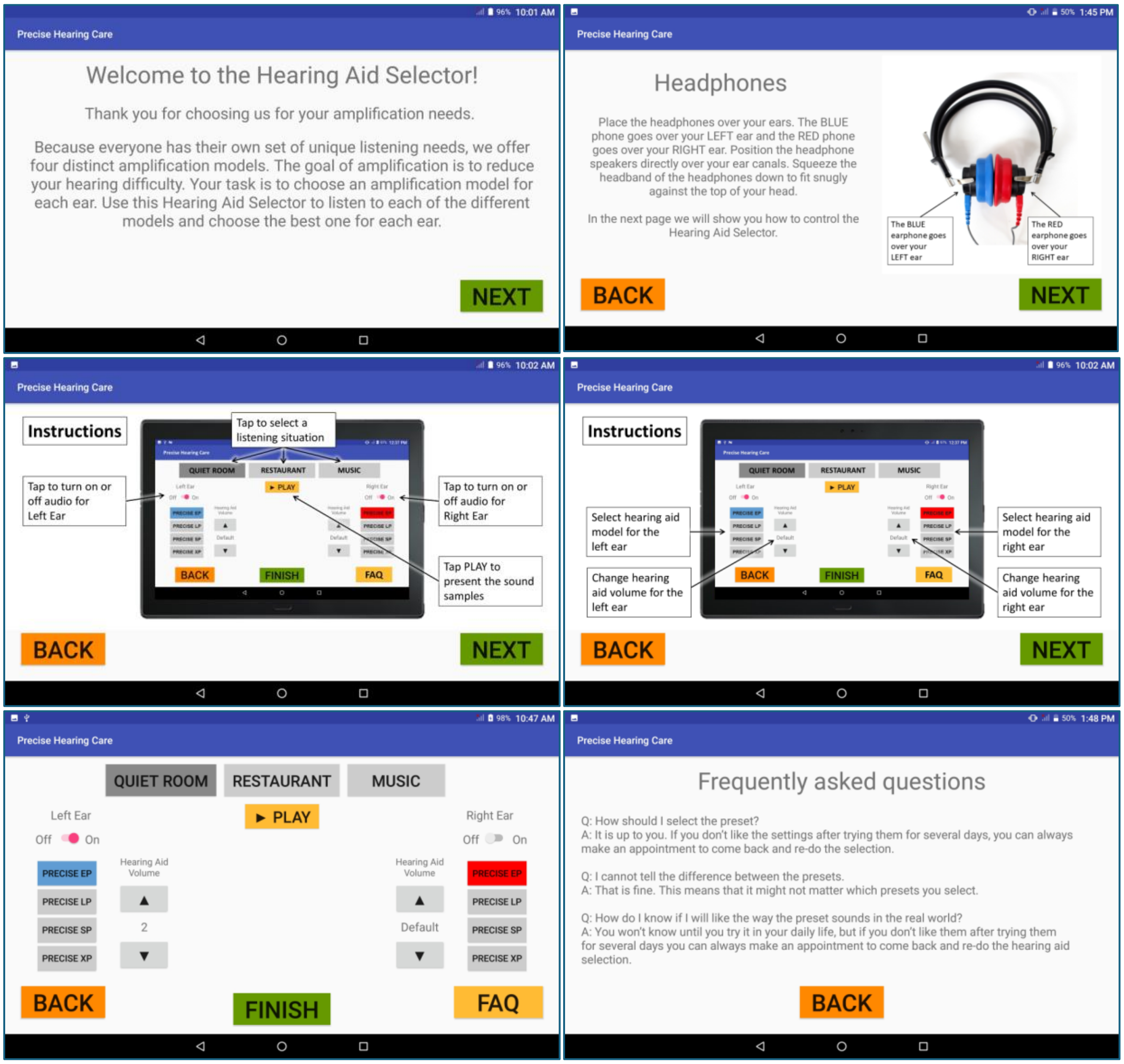

eTable 2. Differences, as Described by the Manufacturer, Between High-End and Low-End Hearing Aids

Feature	High-end (retail price per pair ≈ $4,400 in 2018)	Low-end (retail price per pair ≈ $1,100 in 2018)
Signal processing channels	48	12
Hearing programs	6	4
Extended dynamic range	Yes	No
Extended bandwidth	Yes	No
Directionality	Automatic/Adaptive	Automatic/Fixed
Narrow directionality	Yes	No
General-purpose noise reduction	Yes	Yes
Impulse noise reduction	Yes	No
Spatial noise reduction	Yes	No
Reverberation reduction	Yes	No
Wind noise reduction	Yes	No
Feedback cancellation	Yes	Yes
Smartphone app	Yes	Yes
Volume control	Yes	Yes
Program control	Yes	Yes
Gain-frequency response adjustment	Yes	No
Directionality control (direction and width of the beam)	Yes	No

eMethods 2. Glasgow Hearing Aid Benefit Profile as an Ecological Momentary Assessment Survey: Implementation, Processing, and Analysis

The primary outcome measure of the study—the Glasgow Hearing Aid Benefit Profile (GHABP)[4]—was administered as a smartphone-based ecological momentary assessment (EMA) survey. EMA involves repeatedly asking respondents to report their experiences during or shortly after the experiences in natural environments.[5]

Implementation

We implemented EMA using an application, AudioSense,[6] on Moto G6 Play smartphones owned by the laboratories. The original GHABP was modified to make it suitable for EMA (see **eTable 3** below). At the start of each EMA survey, the app first asked if a predefined listening situation from the GHABP had occurred in the past 1.5 hours. Participants responded by tapping "Yes" or "No" on the smartphone screen. If the response was "No," the app would present the next predefined situation. The GHABP includes four predefined listening situations: TV listening, small conversation in quiet, conversation in noise, and group conversation (**eTable 3**).

If the response was "Yes," the app would present two questions during pre-fitting assessments (initial hearing disability and initial hearing handicap) and five questions during post-fitting assessments (HA use, HA benefit, residual hearing disability, residual hearing handicap, and HA satisfaction). Importantly, in the post-fitting assessment, if participants indicated in the HA Use question that they had not used HAs in the past 1.5 hours ("Never/Not at all"), the subsequent questions on HA benefit, residual hearing disability, residual hearing handicap, and HA satisfaction were not presented.

Both pre-fitting and post-fitting assessments lasted 7 days. During the 7-day assessments, participants carried the study smartphones with them as they engaged in their daily activities. The EMA app triggered survey prompts at random intervals, roughly every 1.5 hours, within a time frame specified by each participant. Participants completed the questionnaire on the smartphones, reflecting on their listening experiences over the previous 1.5 hours. Participants were encouraged to respond to as many surveys as possible, provided it was safe and convenient (e.g., not while driving). If a survey was skipped or missed, participants were instructed to wait for the next notification. Although the app allowed for the initiation of surveys, participants were advised to do so only if a survey had crashed or if they accidentally declined it.

Processing

Survey responses were coded as numerical values ranging from 1 to 5, with higher values indicating better outcomes, following the recommendations of the GHABP authors.[4] The pre-fitting score was calculated as the average of the two items assessing initial hearing disability and handicap across the four predefined listening situations. For post-fitting assessments, the adaptive nature of the EMA question presentation—where subsequent items were skipped if the response to the initial HA Use question was "Never/Not at all"—makes it unsuitable to calculate a global score by averaging across all items. Therefore, two primary outcome scores were developed: (1) the EMA-GHABP Use score, calculated as the average of the HA Use question across the four listening situations, and (2) the EMA-GHABP Global score, derived from the average of the remaining four items (HA benefit, residual hearing disability, residual hearing handicap, and HA satisfaction) across the four listening situations. These calculations were performed for each EMA survey in both pre-fitting and post-fitting assessments.

Analysis

Since previous research has shown that EMA-GHABP Use score data are often right-skewed,[7] the post-fitting EMA-GHABP Use score was dichotomized for analysis as planned a priori (1: using HAs "all the time"; 0: otherwise). A generalized linear mixed model with a logit link function was used to estimate the probability of reporting HA use "all the time," with the dichotomized Use score from each EMA survey as the dependent variable. For analyzing the post-fitting EMA-GHABP Global score, a linear mixed model was used, with the post-fitting EMA-GHABP Global score from each EMA survey as the dependent variable. The pre-fitting EMA-GHABP scores averaged across all EMA surveys completed by each participant were controlled for in the statistical model.

For both models, study site (University of Iowa vs. Vanderbilt University Medical Center) was included as a covariate. A conservative Tukey adjustment was applied for pairwise comparisons between AUD, OTC+, and OTC.

Since the Use score and Global score—both derived from the primary outcome measure (EMA-GHABP)—reflect HA usage and HA benefit/satisfaction, respectively, they represent two distinct dimensions of patient outcomes.[8]

Therefore, following the recommendation of Cao and Zhang,[9] separate analyses were conducted for these two variables without additional alpha adjustment.

In EMA research, the number of completed EMA surveys often varies considerably across participants. While data from participants with fewer surveys might be considered less reliable, the linear mixed model appropriately accounts for this variability. Specifically, the model accommodates differences in the number of completed EMA surveys by assigning less weight to participants with fewer observations. It assumes that any missing responses are Missing At Random and that those missing values would be consistent with that individual's observed values. Thus, the model incorporates and accounts for the known characteristics of all participants. Given this, and the lack of a widely accepted standard for handling low EMA survey completion rates,[10] we predetermined that all participants would be included in the analysis, without excluding those with a low number of completed surveys.

© 2025 Wu Y-H et al. *JAMA Otolaryngology–Head & Neck Surgery*.

eTable 3. EMA-GHABP Questions and Response Options. Square brackets show the question topic.

Questions	Response options
Listening Situation	
Did this situation happen in the past 1.5 hours?	□ Yes<br>□ No
**[Situation 1]** Listening to the TV<br>**[Situation 2]** Having a conversation with one other person when there is no background noise<br>**[Situation 3]** Carrying on a conversation in a busy street, a store, or other noisy environment<br>**[Situation 4]** Having a conversation with several people in a group	
Pre-fitting Assessment (unaided)	
**[Initial Hearing Disability]** In this situation, how much difficulty did you have?	□ No difficulty<br>□ Only slight difficulty<br>□ Moderate difficulty<br>□ Great difficulty<br>□ Cannot manage at all
**[Initial Hearing Handicap]** In this situation, how much did any difficulty in this situation worry, annoy or upset you?	□ Not at all<br>□ Only a little<br>□ A moderate amount<br>□ Quite a lot<br>□ Very much indeed
Post-fitting Assessment (aided)	
**[Hearing Aid Use]** In this situation, what proportion of the time did you wear the hearing aids?	□ Never/Not at all<br>□ About ¼ of the time<br>□ About ½ of the time<br>□ About ¾ of the time<br>□ All the time
**[Hearing Aid Benefit]** In this situation, how much did the hearing aids (HA) help you?	□ HAs no use at all<br>□ HAs are some help<br>□ HAs are quite helpful<br>□ HAs are a great help<br>□ Hearing is perfect with HAs
**[Residual Hearing Disability]** In this situation, with the hearing aids, how much difficulty did you have?	□ No difficulty<br>□ Only slight difficulty<br>□ Moderate difficulty<br>□ Great difficulty<br>□ Cannot manage at all
**[Residual Hearing Handicap]** In this situation, with the hearing aids, how much did any difficulty in this situation worry, annoy or upset you?	□ Not at all<br>□ Only a little<br>□ A moderate amount<br>□ Quite a lot<br>□ Very much indeed

© 2025 Wu Y-H et al. *JAMA Otolaryngology–Head & Neck Surgery*.

**[Hearing Aid Satisfaction]** For this situation, how satisfied were you with the hearing aids?	Not satisfied at all<br>A little satisfied<br>Reasonably satisfied<br>Very satisfied<br>Delighted with hearing aids

© 2025 Wu Y-H et al. *JAMA Otolaryngology–Head & Neck Surgery*.

eFigure 2. Mean post-fitting, as-worn real-ear aided response of the three service models (top figure) and the two technology levels (bottom figure), along with the mean targets prescribed by NAL-NL2[11] for a 65 dB SPL speech input level, for 245 participants who completed the study.

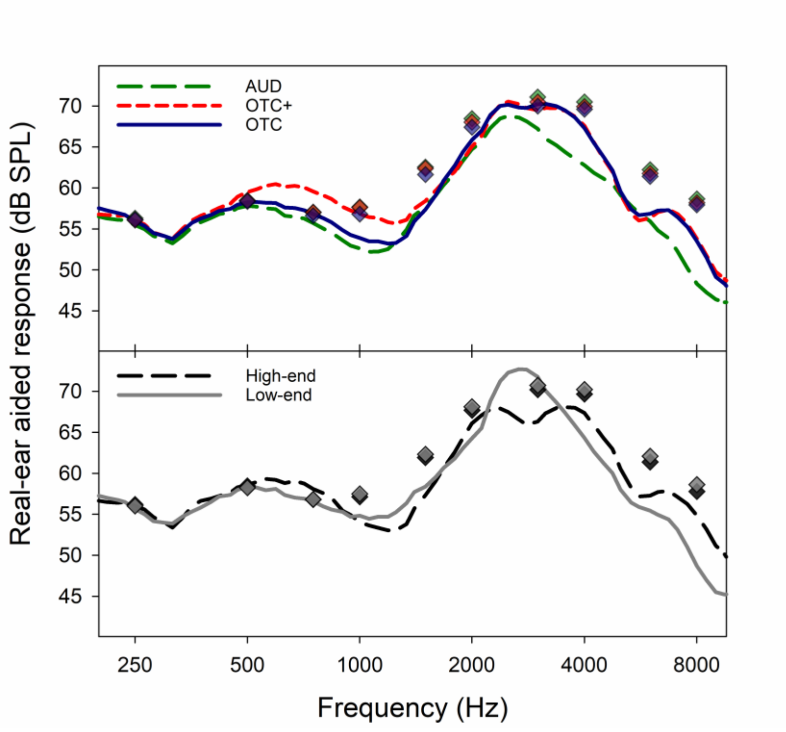

© 2025 Wu Y-H et al. *JAMA Otolaryngology–Head & Neck Surgery*.

eTable 4. Number (%) of Participants Requesting Follow-Up Laboratory Visits. Participants in the AUD group were allowed to request follow-up visits with audiologists as needed, in addition to the mandatory one-week follow-up appointment. Participants in the OTC+ group were permitted up to two brief follow-up visits with audiologists to address issues related to their hearing aids. Participants in the OTC group could request laboratory visits exclusively for the purpose of reselecting presets.

	High-end	Low-end
AUD	7 (16.3)	5 (12.5)
OTC+	9 (22.0)	7 (17.9)
OTC	4 (9.5)	6 (15.0)

© 2025 Wu Y-H et al. *JAMA Otolaryngology–Head & Neck Surgery*.

eTable 5. Effect Sizes of Pairwise Comparisons. The effect size for the EMA-GHABP Global score was calculated by dividing the estimated score difference of each contrast by the square root of the sum of the variances of the random effects in the linear mixed model. For all other outcome measures, the effect size was determined by dividing the estimated score difference of each contrast by the residual standard error of the regression models. The resulting effect size approximates Cohen's *d*. Effect sizes were not calculated for the Use scores of the EMA-GHABP and the Retro-GHABP because these outcomes were analyzed using a generalized linear mixed model with a logit link function and a logistic regression, respectively.

	AUD vs OTC+	AUD vs OTC	OTC+ vs OTC	High-end vs Low-end
EMA-GHABP, Global score	0.57	0.54	0.03	0.04
Retro-GHABP, Global score	0.68	0.52	0.16	0.13
PHAP	0.18	0.18	0.004	0.09
HHIE/A	0.35	0.36	0.02	0.12
SADL	0.59	0.68	0.09	0.13
CST	0.27	0.04	0.23	0.21

© 2025 Wu Y-H et al. *JAMA Otolaryngology–Head & Neck Surgery*.

eReferences

1. Urbanski D, Hernandez H, Oleson J, Wu Y-H. Toward a new evidence-based fitting paradigm for over-the-counter hearing aids. *American Journal of Audiology*. 2021;30(1):43-66.
2. Venkitakrishnan S, Urbanski D, Wu Y-H. Efficacy and Effectiveness of Evidence-Based Non–Self-Fitting Presets Compared to Prescription Hearing Aid Fittings and a Personal Sound Amplification Product. *American Journal of Audiology*. 2024;33(1):31-54.
3. Bailey A. Full List of OTC Hearing Aids in 2024. Accessed August 26, 2024. https://www.hearingtracker.com/otc-hearing-aids/full-list?q=otc
4. Gatehouse S. Glasgow Hearing Aid Benefit Profile: Derivation and validation of a client-centered outcome measure for hearing aid services. *Journal of the American Academy of Audiology*. 1999;10(02):80-103.
5. Shiffman S, Stone AA, Hufford MR. Ecological momentary assessment. *Annual Review of Clinical Psychology*. 2008;4(1):1-32.
6. Hasan SS, Lai F, Chipara O, Wu Y-H. AudioSense: Enabling real-time evaluation of hearing aid technology in-situ. *Proceedings of the 26th IEEE International Symposium on Computer-Based Medical Systems*. IEEE; 2013:167-172.
7. Wu Y-H, Stangl E, Chipara O, Gudjonsdottir A, Oleson J, Bentler R. Comparison of in-situ and retrospective self-reports on assessing hearing aid outcomes. *Journal of the American Academy of Audiology*. 2020;31(10):746-762.
8. Humes LE. Modeling and predicting hearing aid outcome. *Trends in Amplification*. 2003;7(2):41-75.
9. Cao J, Zhang S. Multiple comparison procedures. *JAMA*. 2014;312(5):543-544.
10. Schinkel-Bielefeld N, Burke L, Holube I, et al. Implementing ecological momentary assessment in audiological research: Opportunities and challenges. *American Journal of Audiology*. 2024;33(3):648-673.
11. Keidser G, Dillon H, Flax M, Ching T, Brewer S. The NAL-NL2 prescription procedure. *Audiology Research*. 2011;1(1):e24.

© 2025 Wu Y-H et al. *JAMA Otolaryngology–Head & Neck Surgery*.
