# Supplementary material for: Hearing Aid Service Models, Technology, and Patient Outcomes: A Randomized Clinical Trial
Source: JAMA Otolaryngol Head Neck Surg. 2025 May 15;151(7):684–92. doi: 10.1001/jamaoto.2025.1008 (PMC12082484; doi:10.1001/jamaoto.2025.1008)
Supplement: Supplement 3. — Data sharing statement [file jamaotolaryngolheadnecksurg-e251008-s003.pdf]

Data Sharing Statement

Wu. Hearing Aid Service Models and Technology and Patient Outcomes. *JAMA Otolaryngol Head Neck Surg*. Published May 15, 2025. doi:10.1001/jamaoto.2025.1008

Data
Additional Information: ClinicalTrials.gov Identifier: NCT03579563
Data available: Yes
Data types: Deidentified participant data
How to access data: The de-identified raw data supporting the conclusions of this article will be available upon request by contacting the corresponding author at yu-hsiang-wu@uiowa.edu.
When available: With publication

Supporting Documents
Document types: None

Additional Information
Who can access the data: The data will be made available to researchers whose proposed use has been approved.
Types of analyses: for any purpose
Mechanisms of data availability: after approval of a proposal and with a signed data access agreement
